# Late HIV/AIDS diagnosis among people living with HIV in Wuhan in 2023

**DOI:** 10.3389/fmicb.2025.1594847

**Published:** 2025-05-21

**Authors:** Li Tang, Yan-Tao Du, Wen-Hua Kong, Pan Liu, Ze-Rong Zhu, Shi-Zhe Xie, Man-Qing Liu

**Affiliations:** Department of Pathogen and Immunology, Wuhan Center for Disease Control and Prevention, Wuhan, China

**Keywords:** late HIV diagnosis, advanced HIV diseases, CD4 counts, men who have sex with men, Wuhan

## Abstract

Late HIV diagnosis is defined as presenting for care with a CD4 count <350 cells/μl or an AIDS-defining event, and it continues to be a significant challenge in the global effort to prevent and control HIV/AIDS. To examine the late diagnosis of HIV in Wuhan, we retrospectively analyzed cases of late diagnosis among newly identified people living with HIV in 2023. Of the 383 newly diagnosed individuals with HIV infection, 260 (67.89%) were diagnosed late and their CD4 counts were less than 350 cells/μl. Among them, 135 (35.25%) had advanced HIV disease, with CD4 counts below 200 cells/μl. Compared to those diagnosed promptly, the population with late HIV diagnosis had a higher viral load, older age, and lower CD/CD8 ratio. They were also more likely to be men who have sex with men (MSM) or farmers, and were typically diagnosed through voluntary counseling and testing (VCT) or clinical patients. These findings highlight the high rate of late HIV diagnosis in Wuhan, suggesting the need for more attention and more targeted measures toward earlier diagnosis within the population.

## Introduction

Human immunodeficiency virus (HIV) infection continues to be a major global public health issue, having claimed 42.3 million lives so far (World Health Organization, 2024a). HIV targets the body’s immune system, specifically CD4 cells, weakening immunity against opportunistic infections, such as bacterial, fungal, and viral infections (Meintjes and Maartens, 2024; Ji et al., 2024; You et al., 2023; Jose-Abrego et al., 2023; Changizi et al., 2023; Gu et al., 2024; Oranuka et al., 2024; Baghi et al., 2024), and contributing to the development of certain cancers (Baghi et al., 2024; Ding et al., 2023; Vulchi et al., 2023; Chudasma et al., 2023; Zhang et al., 2023; Omar et al., 2024) and other diseases (Nwabuko, 2023; Hudson et al., 2024; Plummer and Pavia, 2021). As a result, HIV caused the deaths of 630,000 people in the world in 2023 (World Health Organization, 2024a). Meanwhile, HIV infection often weakens immune function, resulting in vaccine immunization failure (Plummer and Pavia, 2021; Bello et al., 2024; Cheung et al., 2023). Fortunately, the widespread use of effective antiretroviral therapy (ART) has dramatically reduced HIV-related morbidity, mortality, and transmission (Zhou et al., 2014), transforming acquired immune deficiency syndrome (AIDS) into a manageable chronic disease (Luo et al., 2023; Chakrabarti and Chattopadhyay, 2024).

Testing for HIV is the only way to know if a person is infected. For this reason, the Joint United Nations Programme on HIV/AIDS (UNAIDS) has identified the detection of HIV/AIDS as the primary goal of the 95–95-95 target by 2025 (Mine et al., 2024). Despite many measures taken worldwide to promote testing, only 86% of all people living with HIV knew their HIV status, and only 1.3 million new diagnoses were reported in 2023 (UNAIDS, 2024), with more than half of those diagnoses being made late (World Health Organization, 2024b). In China, despite the implementation of a series of policies such as the “Four Frees and One Care” (Liu et al., 2013) and the establishment of a highly sophisticated HIV surveillance and reporting system (Cai et al., 2024), an average of 15 new HIV infections were identified every hour in 2021 (Wang et al., 2022). Additionally, the rate of late HIV diagnosis in China ranged from 35.5 to 70.2% (Hu et al., 2019). According to the definition endorsed by the European Centre for Disease Prevention and Control (ECDC) and the World Health Organization (WHO), late diagnosis, rather than late presentation, is defined as having a CD4 count <350 cells/μl or an AIDS-defining event (Croxford et al., 2022). Late HIV diagnosis has been shown to be associated with poor outcomes, an increased risk of ongoing HIV transmission, high healthcare costs, and a significant impact on long-term health (Collins et al., 2022). Considering that the proportion of late diagnoses was affected by regions, populations, and policies, we conducted this retrospective study of late HIV diagnoses in Wuhan to guide the prevention and control of HIV/AIDS in the region.

## Methods

### Data collection

As described previously (Liu et al., 2013), individuals who tested HIV antibody positive through Western blot (WB) were followed up with laboratory testing for CD4 + T cell count and plasma HIV-1 viral load. The laboratory testing and follow-up of people living with HIV were reviewed and approved by the Ethics Committee of the Wuhan Center for Disease Prevention and Control, and verbal informed consent was obtained from patients. The epidemic information and laboratory results of individuals were stored in the Managing Database of HIV/AIDS in Wuhan. Thus, data from this database were collected, including demographic information, sampling dates, and laboratory results. The inclusion criteria were: (1) newly diagnosed as HIV antibody positive in 2023 and (2) having a CD4 + T cell count test within 3 months of the HIV diagnosis date. The exclusion criteria were: (1) previously diagnosed as HIV antibody positive and (2) having the first CD4 + T cell count test conducted more than 3 months after the HIV diagnosis date. Based on their CD4 counts, individuals with CD4 < 350 cells/μl were classified as having a late HIV diagnosis, and those with CD4 < 200 cells/μl were described as having advanced HIV disease.

### Statistical analysis

Categorical variables were analyzed using the chi-squared of Fisher’s exact test, while continuous variables were analyzed using student *t*-tests. Statistical analysis was performed using GraphPad Instat version 9.0.0 (GraphPad Software, San Diego, CA), and data were presented as mean ± standard deviation (SD). A *p*-value of <0.05 was considered statistically significant.

## Results

**Figure 1:**
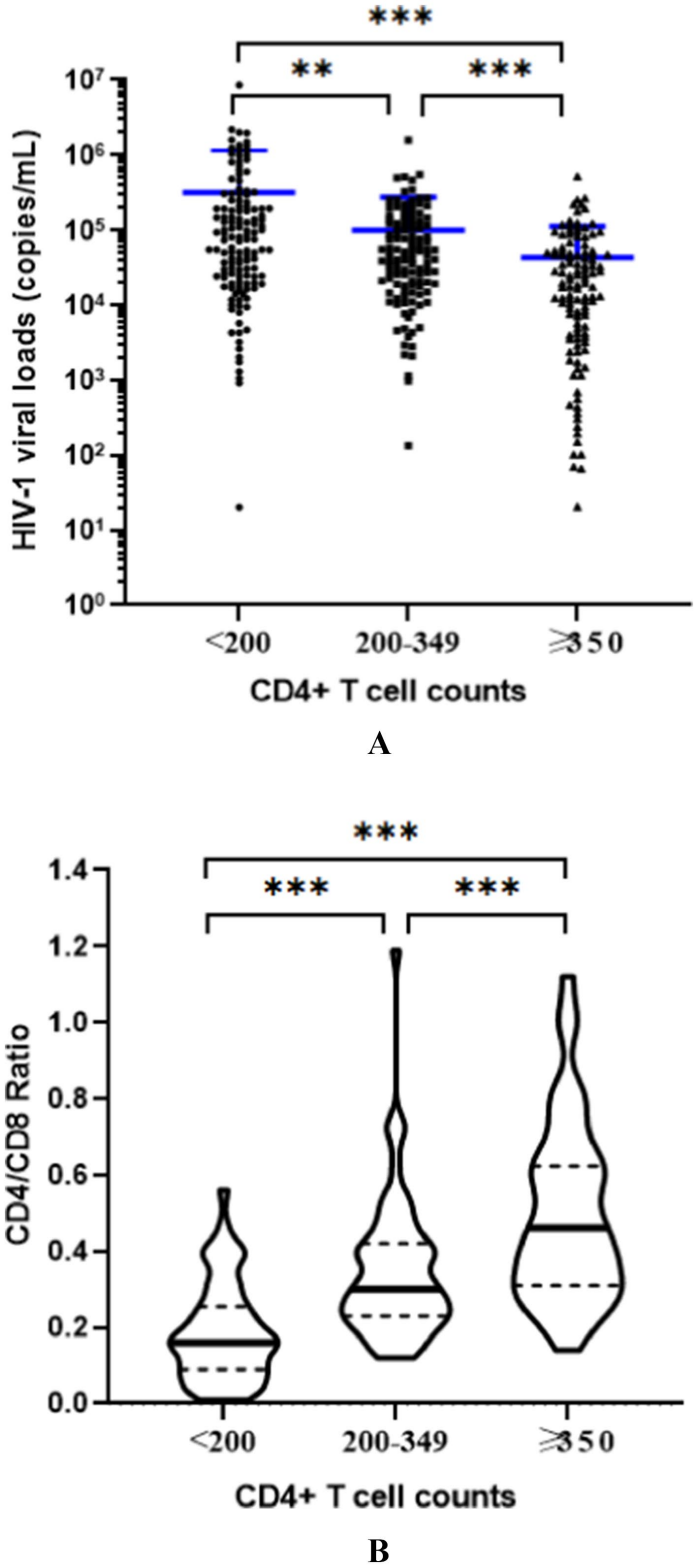
Comparison of HIV-1 viral loads **(A)** and CD4/CD8 ratios **(B)** in the newly identified subjects with different CD4 + T cell counts. The results indicated that, compared to those diagnosed promptly (CD4 + T cell counts ≥ 350/μl), the subjects with late diagnosis (CD4 + T cell counts < 350/μl) exhibited both higher HIV-1 viral load and lower CD4/CD8 ratios (*p* < 0.01), with those with advanced HIV disease (AHD, CD4 + T cell counts <200/μl) demonstrating even more pronounced abnormalities in these parameters. *p < 0.05, **p < 0.01, ***p < 0.001.

In 2023, a total of 383 individuals newly diagnosed with HIV-1 infection were enrolled, including 337 men (87.99%) and 46 women. The median age was 38 years [interquartile range (IQR): 24–53 years]. Men (36.52 ± 15.76 years) were significantly younger than women (51.54 ± 10.86 years; *p* < 0.0001). Based on CD4 + T cell counts tested within 3 months of diagnosis, 135 individuals (35.25%) had CD4 + T cell counts <200 cells/μl, while 125 (32.64%) and 123 (32.11%) individuals had counts of 200–349 cells/μl and ≥350 cells/μl, respectively (Table 1). Thus, according to the definition, the rates of late HIV diagnosis (CD4 < 350 cells/μl) and advanced HIV disease (AHD) (CD4 < 200 cells/μl) were 67.89 and 35.25%, respectively, in Wuhan city in 2023. Subjects with late HIV diagnosis had CD4 + T cell counts of 186.60 ± 96.24 cells/μl, CD8 + T cell counts of 887.48 ± 494.48 cells/μl, CD4/CD8 value of 0.27 ± 0.18, and HIV-1 viral load of 2.13 ± 6.26 × 10^5^ copies/ml. In comparison, patients with advanced HIV disease (AHD) had CD4+, CD8 + T cells counts, CD4/CD8 ratio, and viral load for the patients with AHD were 110.24 ± 62.77 cells/μl, 790.83 ± 559.02 cells/μl, 0.19 ± 0.13, and 3.16 ± 8.40 × 10^5^ copies/ml, respectively (Table 1). Compared to those diagnosed promptly, the subjects with late diagnosis exhibited both higher HIV-1 viral load and lower CD4/CD8 ratios (*p* < 0.01), with those with AHD demonstrating even more pronounced abnormalities in these parameters (Figure 1).

**Table 1:** Laboratory results of newly diagnosed HIV/AIDS in Wuhan in 2023.

Variables	CD4 < 200cells/μl		CD4 200–349 cells/μl		CD4 ≥ 350 cells/μl		Total	
	n	Value^*^	n	Value^*^	n	Value^*^	n	Value^*^
n	135	35.25%	125	32.64%	123	32.11%	383	100%
CD4 + T cell counts	135	110.24 ± 62.77	125	269.06 ± 43.40	123	490.79 ± 144.71	383	284.29 ± 182.19
CD8 + T cell counts	54	790.83 ± 559.02	65	967.77 ± 421.42	90	1185.44 ± 533.97	209	1015.79 ± 531.59
CD4/CD8 ratio	54	0.19 ± 0.13	65	0.35 ± 0.18	90	0.49 ± 0.23	209	0.37 ± 0.23
HIV-1 viral load *10^5^	135	3.16 ± 8.40	124	1.01 ± 1.72	120	0.43 ± 0.69	380	1.60 ± 5.25

*n*, number.

* Value was presented as percentage or mean ± standard deviation.

### Factors associated with late HIV diagnosis

**Figure 2:**
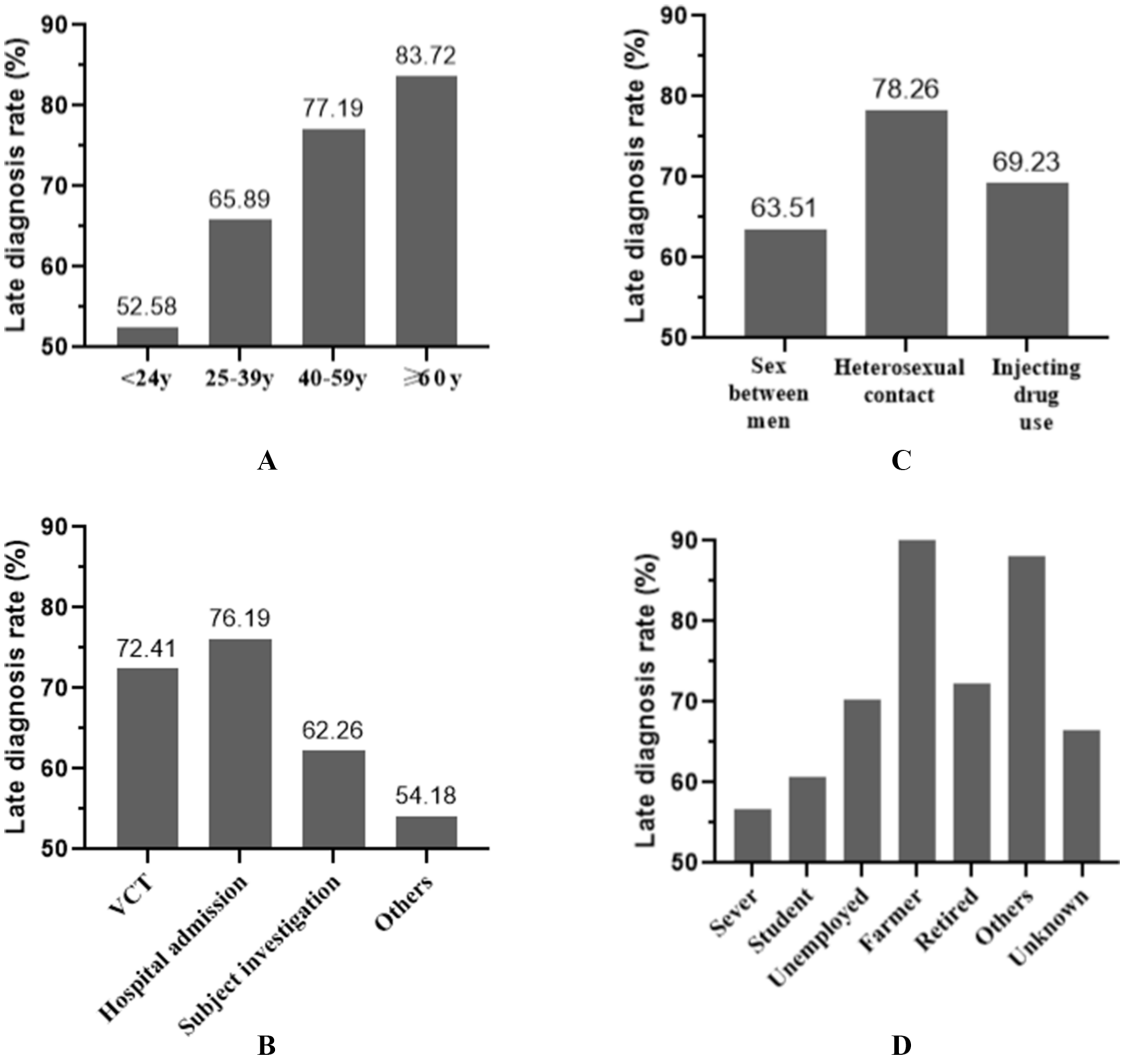
Comparison of late HIV diagnosis rate among the newly identified people living with HIV classified by age groups **(A)**, diagnosis routes **(B)**, infection routes **(C)**, or occupation **(D)**. The highest late HIV diagnosis rate was primarily to be older than 60 years, farmers, infected by heterosexual contact, diagnosed through subject investigation.

In Table 2, we compared various factors between patients with late diagnosis and those diagnosed promptly. Significant differences were observed in age, HIV diagnosis routes, and occupations between the two groups (*p* < 0.05). Specifically, patients with late diagnosis were significantly older (40.73 ± 16.23 years) than those diagnosed promptly (33.22 ± 14.31 years, *p* < 0.001). Among all age groups, patients older than 60 years had the highest rate of late HIV diagnosis rate (83.72%, Figure 2A). Individuals with late diagnosis were mainly men who have sex with men (MSM, 54.65%) and were diagnosed through voluntary counseling and testing (VCT, 16.15%) or as clinical patients (49.23%). The rates of late diagnosis among those diagnosed through VCT and clinical settings were 72.41 and 76.19%, respectively (Figure 2B). However, MSM had the lowest late HIV diagnosis rate (63.51%, Figure 2C) compared to individuals infected through heterosexual intercourse or injection drug use. When classified by occupation, farmers had the highest rate of late HIV diagnosis (100%, Figure 2D).

**Table 2:** Comparison of characteristics between late HIV diagnosis and those diagnosed promptly.

Variables	Late HIV diagnosis (CD4 < 350 cells/μl)	HIV diagnosed promptly (CD4 ≥ 350 cells/μl)	*p*
*n*	260 (67.89%)	123 (32.11%)	
Sex			0.2405
Male	225 (86.54%)	112 (91.006%)	
Female	35 (13.46%)	11 (8.94%)	
Age			0.0002
<24y	51 (19.61%)	46 (37.40%)	
25–39y	85 (32.69%)	44 (35.77%)	
40–59y	88 (33.85%)	26 (21.14%)	
≥60y	36 (13.85%)	7 (5.69%)	
Mean (years)	40.73 ± 16.23	33.22 ± 14.31	<0.0001
Ethnicity			0.1271
Han	254 (97.69%)	116 (94.31%)	
Others	6 (2.31%)	7 (5.69%)	
Marital status			0.0874
Married	67 (25.77%)	23 (18.70%)	
Single	119 (45.77%)	73 (59.35%)	
Divorced or widowed	27 (10.38%)	8 (6.50%)	
Unknown	47 (18.08%)	19 (15.45%)	
Main transmission risk			0.0055
MSM	141 (54.23%)	81 (65.85%)	
Heterosexual intercourse	90 (34.62%)	25 (20.33%)	
IDU	27 (10.38%)	12 (9.76%)	
Unknown	2 (0.77%)	5 (4.06%)	
HIV diagnosis through			0.0103
VCT	42 (16.15%)	16 (13.01)	
Clinical patients	128 (49.24%)	40 (32.52%)	
Subject investigation	33 (12.69%)	20 (16.26%)	
Voluntary blood donation	4 (1.54%)	3 (2.44%)	
Physical examination^*^	4 (1.54%)	7 (5.69%)	
Positive patient’s spouse	3 (1.15%)	2 (1.63%)	
Others	46 (17.69%)	35 (28.46%)	
Occupation			0.0011
Commercial services	47 (18.08%)	36 (29.27%)	
Students	31 (11.92%)	20 (16.26%)	
Household or unemployment	26 (10.00%)	11 (8.94%)	
Farmers	15 (5.77%)	0 (0)	
Retired	13 (5.00%)	5 (4.06%)	
Others	37 (14.23%)	5 (4.06%)	
Unknown	91 (35.00%)	46 (37.40%)	

n, number; VCT, voluntary counseling and testing; IDU, injection drug users; MSM, men who have sex with men.

* Physical examination for recruits, entry-exit personnel, prisoners, maternal, recipient of blood, etc.

## Discussion

In this study, we reported that the rate of late HIV diagnosis in Wuhan city in 2023 was 67.89%, with 35.25% of cases classified as advanced HIV disease. Although this conclusion was based only on laboratory CD4 counts and lacked clinical evidence (Croxford et al., 2022), it appears similar to those previously reported in China, such as in Hunan province (66.6%) (Su et al., 2024), Guangxi Zhuang Autonomous Region (70.2%) (Hu et al., 2019), and Suzhou city (57.6%) (Xu et al., 2023). A study spanning more than 10 years revealed that late HIV diagnosis in China had steadily increased in recent years (Shi et al., 2022). Despite huge efforts to improve testing, the high rate of late HIV diagnosis remains a great challenge to achieving the “95–95-95” targets proposed by UNAIDS.

Many factors affect the late diagnosis of HIV infection, including demographic characteristics and socio-economic factors, testing awareness and psychological factors, medical resources and testing routes, infection and transmission routes, policies, and prevention and control strategies (Collins et al., 2022; Chopel et al., 2014; Sun et al., 2021). In this study, we found that the population with a late HIV diagnosis differs from those diagnosed promptly in terms of age, infection route, testing route, and occupation. Specifically, the proportion of late HIV diagnoses in Wuhan was highest among individuals over 60 years, those infected through heterosexual transmission, farmers, and clinical patients. The high rate of late HIV diagnosis among the elderly and farmers may be associated with their lower awareness of AIDS-related knowledge and active testing. Interestingly, our research suggested that the proportion of late diagnoses increased with age, especially for the age group of ≥60 years (83.72%). This finding is consistent with previous reports in China and may be related to factors such as living conditions, awareness of testing, and physical conditions (Zheng, 2020). Similarly, the higher rate of late HIV diagnosis among farmers may also be related to their awareness and accessibility of HIV testing. Late HIV diagnosis among heterosexual infected individuals was relatively higher than that of MSM or IDU. In recent years, several intervention programs to promote testing frequency, the use of PrEP, testing adherence, and heightened awareness have been conducted among MSM, which may influence the proportion of late diagnoses. In addition, Table 1 indicates that individuals who were divorced or widowed had a higher risk of late HIV diagnosis than other marital status groups, which may be related to factors such as lack of partner support or delayed medical treatment due to psychological pressure. Therefore, it is crucial to implement HIV/AIDS-related health education for a broader population and conduct HIV screening for the entire population, especially key groups such as the elderly and farmers.

HIV viral load and the CD4/CD8 ratio are important measures in monitoring HIV infection. The HIV-1 viral load steadily increases and the risk of transmission 3–5 years after infection is also higher in the late HIV diagnosis population (Collins et al., 2022). Our research showed that the HIV-1 viral load in individuals with late HIV diagnosis was significantly higher than that in those diagnosed promptly, with the highest viral loads observed in those with advanced HIV disease. This finding is consistent with our previous research, which found a negative correlation between viral load and CD4 + T cell counts (Liu et al., 2013). Late HIV diagnosis and high viral load both increase the risk of HIV transmission and pose significant challenges to HIV/AIDS prevention and control. The CD4/CD8 ratio was an important indicator for evaluating immune status. People with late HIV diagnosis typically have lower CD4 cell counts and higher CD8 cell counts, which results in a significantly lower CD4/CD8 ratio than the normal range. This study indicated that the CD4/CD8 ratio was positively correlated with CD4 + T cell count, with individuals who were diagnosed later having lower CD4/CD8 ratios. Therefore, early diagnosis and treatment would help to improve the immune status of individuals and reduce the spread of HIV.

Since the first case of HIV was reported in China, several key policies have been implemented to prevent HIV, including the Blood Donation Law (1998), the first Five-Year Action Plan for the Containment and Control of HIV/AIDS (2001), the “Four Frees and One Care” policy (2003), and the “Five Expands, Six Strengths” Strategy (2010) (Lu et al., 2020). Despite significant progress towards achieving the UNAIDS “95–95-95” targets by 2025, especially for the detection of HIV/AIDS, late HIV diagnosis as a key metric to measure the public health response, remains stubbornly high in nearly every country (Croxford et al., 2022), including China. Thus, a more thorough investigation of late HIV diagnosis and its influencing factors is needed. Such studies will provide insight into the blind spots in HIV/AIDS intervention in the region, helping to propose more targeted intervention measures, including targeted screening, public awareness campaigns (especially for the elderly and farmers), strengthening primary care engagement, and improving the precision of intervention through artificial intelligence or big data-based predictive models.

In summary, this study reported that the rate of late HIV diagnosis in Wuhan in 2023 was as high as 67.89%, influenced by various economic and social factors. This highlights the need for local HIV/AIDS prevention and control efforts to focus not only on identifying more people living with HIV but also on improving early diagnosis of HIV/AIDS.

## Data Availability

The original contributions presented in the study are included in the article/supplementary material, further inquiries can be directed to the corresponding author.

## References

[ref1] Baghi H. B. Aghbash P. S. Rasizadeh R. Poortahmasebi V. Alinezhad F. (2024). Cancers associated with human papillomavirus: an overview of prevalence in Iran and the Middle East. Explor. Res. Hypothesis Med. 9, 115–127. doi: 10.14218/ERHM.2023.00053

[ref2] Bello N. Hudu S. A. Alshrari A. S. Imam M. U. Jimoh A. O. (2024). Overview of hepatitis B vaccine non-response and associated B cell amnesia: a scoping review. Pathogens 13:554. doi: 10.3390/pathogens13070554, PMID: 39057781 PMC11279426

[ref3] Cai C. Tang H. Li D. Qin Q. Chen F. Jin Y.. (2024). Evolution of HIV epidemic and emerging challenges - China, 1989-2023. China CDC Wkly 6, 1251–1256. doi: 10.46234/ccdcw2024.251, PMID: 39698324 PMC11649988

[ref4] Chakrabarti S. K. Chattopadhyay D. (2024). From immune sanctuary to neurological battlefield: the role of neuroimmune cells. Explor. Res. Hypothesis Med. 9, 308–327. doi: 10.14218/ERHM.2024.00026, PMID: 39130623

[ref5] Changizi Z. Kajbaf F. Moslehi A. (2023). An overview of the role of peroxisome proliferator-activated receptors in liver diseases. J. Clin. Transl. Hepatol. 11, 1542–1552. doi: 10.14218/JCTH.2023.00334, PMID: 38161499 PMC10752810

[ref6] Cheung C. K. M. Law K. W. T. Law A. W. H. Law M. F. Ho R. Wong S. H. (2023). Efficacy of vaccine protection against COVID-19 virus infection in patients with chronic liver diseases. J. Clin. Transl. Hepatol. 11, 718–735. doi: 10.14218/JCTH.2022.00339, PMID: 36969905 PMC10037513

[ref7] Chopel A. M. Minkler M. Nuru-Jeter A. Dunbar M. (2014). Social determinants of late stage HIV diagnosis and its distributions among African Americans and Latinos: a critical literature review. J. Health Disparit. Res. Pract. 8:1.

[ref8] Chudasma M. P. Shah S. A. Qureshi M. H. N. Shah N. Shah D. Trivedi R.. (2023). Brief insight on nanovesicular liposomes as drug-delivery carriers for medical applications. J. Explor. Res. Pharmacol. 8, 222–236. doi: 10.14218/JERP.2022.00086, PMID: 39130623

[ref9] Collins S. Namiba A. Sparrowhawk A. Strachan S. Thompson M. Nakamura H. (2022). Late diagnosis of HIV in 2022: why so little change? HIV Med. 23, 1118–1126. doi: 10.1111/hiv.13444, PMID: 36397250

[ref10] Croxford S. Stengaard A. R. Brännström J. Combs L. Dedes N. Girardi E.. (2022). Late diagnosis of HIV: An updated consensus definition. HIV Med. 23, 1202–1208. doi: 10.1111/hiv.13425, PMID: 36347523 PMC10100195

[ref11] Ding X. Zhao Z. Liu S. Zhang J. Zhou Y. Xin Y. (2023). Chronic infection considerations in nonalcoholic fatty liver disease patients. Gene Expr. 22, 192–202. doi: 10.14218/GE.2022.00007

[ref12] Gu T. Zheng C. Y. Deng Y. Q. Yang X. F. Bao W. M. Tang Y. M. (2024). Systematic evaluation of guidelines for the diagnosis and treatment of hepatitis E virus infection. J. Clin. Transl. Hepatol. 12, 739–749. doi: 10.14218/JCTH.2023.00508, PMID: 39130619 PMC11310757

[ref13] Hu X. Liang B. Zhou C. Jiang J. Huang J. Ning C.. (2019). HIV late presentation and advanced HIV disease among patients with newly diagnosed HIV/AIDS in southwestern China: a large-scale cross-sectional study. AIDS Res. Ther. 16:6. doi: 10.1186/s12981-019-0221-7, PMID: 30876476 PMC6420760

[ref14] Hudson J. A. Ferrand R. A. Gitau S. N. Mureithi M. W. Maffia P. Alam S. R.. (2024). HIV-associated cardiovascular disease pathogenesis: An emerging understanding through imaging and immunology. Circ. Res. 134, 1546–1565. doi: 10.1161/CIRCRESAHA.124.323890, PMID: 38781300

[ref15] Ji F. Tran S. Ogawa E. Huang C. F. Suzuki T. Wong Y. J.. (2024). Real-world effectiveness and tolerability of interferon-free direct-acting antiviral for 15,849 patients with chronic hepatitis C: a multinational cohort study. J. Clin. Transl. Hepatol. 12, 646–658. doi: 10.14218/JCTH.2024.00089, PMID: 38993510 PMC11233980

[ref16] Jose-Abrego A. Roman S. Rebello Pinho J. R. Gomes-Gouvêa M. S. Panduro A. (2023). High frequency of antiviral resistance mutations in HBV genotypes A2 and H: multidrug resistance strains in Mexico. J. Clin. Transl. Hepatol. 11, 1023–1034. doi: 10.14218/JCTH.2022.00135S, PMID: 37577226 PMC10412697

[ref17] Liu M. Q. Tang L. Kong W. H. Zhu Z. R. Peng J. S. Wang X.. (2013). CD4+ T cell count, HIV-1 viral loads and demographic variables of newly identified patients with HIV infection in Wuhan, China. J. Med. Virol. 85, 1687–1691. doi: 10.1002/jmv.23627, PMID: 23861066

[ref18] Lu F. Xu P. McGoogan J. M. Chen W. Ma L. (2020). “Evolution of HIV/AIDS Policy,” in HIV/AIDS in China. eds. Wu Z. Wang Y. Detels R. Bulterys M. McGoogan J. (Singapore: Springer)., PMID:

[ref19] Luo Y. Chen Z. Li Z. Luo A. Zeng Y. Chen M.. (2023). TDF promotes glycolysis and mitochondrial dysfunction to accelerate lactate accumulation by downregulating PGC1α in mice. J. Clin. Transl. Hepatol. 11, 998–1002. doi: 10.14218/JCTH.2022.00082, PMID: 37408811 PMC10318276

[ref20] Meintjes G. Maartens G. (2024). HIV-associated tuberculosis. N. Engl. J. Med. 391, 343–355. doi: 10.1056/NEJMra2308181, PMID: 39047241

[ref21] Mine M. Stafford K. A. Laws R. L. Marima R. Lekone P. Ramaabya D.. (2024). Progress towards the UNAIDS 95-95-95 targets in the fifth Botswana AIDS impact survey (BAIS V 2021): a nationally representative survey. Lancet HIV 11, e245–e254. doi: 10.1016/S2352-3018(24)00003-1, PMID: 38467135 PMC11289895

[ref22] Nwabuko O. C. (2023). Multiple myeloma: risk factors, pathogenesis and relationship with anti-myeloma therapies. J. Explor. Res. Pharmacol. 8, 57–65. doi: 10.14218/JERP.2022.00059, PMID: 39130623

[ref23] Omar A. Marques N. Crawford N. (2024). Cancer and HIV: the molecular mechanisms of the deadly duo. Cancers (Basel) 16:546. doi: 10.3390/cancers16030546, PMID: 38339297 PMC10854577

[ref24] Oranuka K. R. Chama C. Adogu I. O. Okafor C. G. Eleje G. U. Ugwu E. O.. (2024). Placental malaria and its relationship with neonatal birth weight among primigravidae: an analytical cross-sectional study. Explor. Res. Hypothesis Med. 9, 181–191. doi: 10.14218/erhm.2023.00015, PMID: 39267914 PMC11391525

[ref25] Plummer M. M. Pavia C. S. (2021). COVID-19 vaccines for HIV-infected patients. Viruses 13:1890. doi: 10.3390/v13101890, PMID: 34696319 PMC8540182

[ref26] Shi L. Tang W. Liu X. Hu H. Qiu T. Chen Y.. (2022). Trends of late HIV presentation and advance HIV disease among newly diagnosed HIV cases in Jiangsu, China: a serial cross-sectional study from 2008 to 2020. Front. Public Health 10:1054765. doi: 10.3389/fpubh.2022.1054765, PMID: 36568791 PMC9773559

[ref27] Su X. Zhong X. Zhang X. Gao Y. Zou X. Chen X.. (2024). Unveiling trends in late diagnosis among 22, 504 people living with HIV in Hunan, China. Sci. Rep. 14:23165. doi: 10.1038/s41598-024-73648-6, PMID: 39369087 PMC11455870

[ref28] Sun C. Li J. Liu X. Zhang Z. Qiu T. Hu H.. (2021). HIV/AIDS late presentation and its associated factors in China from 2010 to 2020: a systematic review and meta-analysis. AIDS Res. Ther. 18:96. doi: 10.1186/s12981-021-00415-2, PMID: 34895254 PMC8665516

[ref29] UNAIDS. (2024). Global HIV & AIDS statistics-Fact sheet. Available online at: https://www.unaids.org/en/resources/fact-sheet (Accessed March, 2025).

[ref30] Vulchi J. Suryadevara V. Mohan P. Kamalanathan S. Sahoo J. Naik D.. (2023). Obesity and metabolic dysfunction-associated fatty liver disease: understanding the intricate link. J. Transl. Gastroenterol. 1, 74–86. doi: 10.14218/JTG.2023.00043

[ref31] Wang H. Tang W. Shang H. (2022). Expansion of PrEP and PEP services in China. Lancet HIV 9, e455–e457. doi: 10.1016/S2352-3018(22)00138-2, PMID: 35688167

[ref32] World Health Organization. (2024a). Data on the size of the HIV epidemic. Available online at: https://www.who.int/data/gho/data/themes/topics/topic-details/GHO/data-on-the-size-of-the-hiv-aids-epidemic?lang=en (Accessed March, 2025).

[ref33] World Health Organization. (2024b). Under-and late diagnosis of HIV is holding back progress to end AIDS in the European Region. Available online at: https://www.who.int/europe/news-room/28-11-2024-under--and-late-diagnosis-of-hiv-is-holding-back-progress-to-end-aids-in-the-european-region (Accessed March, 2025).

[ref34] Xu Z. Shen Q. Wang D. Dong Z. Han W. Tian R.. (2023). Real-world data in late presentation of HIV infection in Suzhou, China: results from four consecutive cross-sectional surveys, 2017-2020. Front. Public Health 11:1084840. doi: 10.3389/fpubh.2023.1084840, PMID: 36895684 PMC9989277

[ref35] You H. Wang F. Li T. Xu X. Sun Y. Nan Y.. (2023). Guidelines for the prevention and treatment of chronic hepatitis B (version 2022). J. Clin. Transl. Hepatol. 11, 1425–1442. doi: 10.14218/JCTH.2023.00320, PMID: 37719965 PMC10500285

[ref36] Zhang W. Du F. Wang L. Bai T. Zhou X. Mei H. (2023). Hepatitis virus-associated non-hodgkin lymphoma: pathogenesis and treatment strategies. J. Clin. Transl. Hepatol. 11, 1256–1266. doi: 10.14218/JCTH.2022.00079S, PMID: 37577221 PMC10412707

[ref37] Zheng Y. J. (2020). Research progress on the influencing factors of late detection of HIV/AIDS cases in China. Acad. J. Guangzhou Med. Univ. 48, 124–127. doi: 10.3969/j.issn.2095-9664.2020.06.32

[ref38] Zhou W. Zhao M. Wang X. Schilling R. F. Zhou S. Qiu H. Y.. (2014). Treatment adherence and health outcomes in MSM with HIV/AIDS: patients enrolled in "one-stop" and standard care clinics in Wuhan China. PLoS One 9:e113736. doi: 10.1371/journal.pone.0113736, PMID: 25438039 PMC4249979

